# Brief Communication: Confocal microscopy of oral streptococcal biofilms grown in simulated microgravity using a random positioning machine

**DOI:** 10.1038/s41526-024-00427-y

**Published:** 2024-09-09

**Authors:** Kelly C. Rice, Ke Aira T. Davis

**Affiliations:** 1 Department of Microbiology and Cell Science, IFAS, University of Florida, Gainesville, FL 32611 USA; 2 Present Address: Health Sciences (Biotechnology), Central Georgia Technical College, Macon, GA 31206 USA

**Keywords:** Microbiology, Biological techniques

## Abstract

Biofilms are a concern for spaceflight missions, given their propensity for biofouling systems and their potential threat to astronaut health. Herein, we describe a random positioning machine-based method for growing fluorescent protein-expressing streptococcal biofilms under simulated microgravity. Biofilms can be subsequently imaged by confocal microscopy without further manipulation, minimizing disruption of architecture. This methodology could be adaptable to other bacteria, potentially standardizing biofilm growth and study under simulated microgravity.

*Streptococcus mutans* is a major contributor to dental caries^1–4^, a microbial shift disease that can be exacerbated by host factors such as decreased saliva flow or a high-sucrose diet. In Skylab mission astronauts, increased recovered counts of oral bacteria (including *Streptococcus sanguinis* and *S. mutans*), decreased salivary lysozyme, and increased dental calculus were reported in response to space flight^5^. Simulated microgravity exposure can also cause increased mandibular and alveolar bone loss^6^ and decreased saliva flow^7^, host factors that could contribute to caries development and/or periodontal disease.

In our previous study of *S. mutans* growth in High Aspect Rotating Vessels (HARVs) using the rotary cell culture system (RCCS), spherical cell aggregates formed in low shear modelled microgravity (LSMMG) when grown in biofilm-promoting medium^8^. These aggregates were more difficult to disrupt compared to cells from normal (1 ×g) gravity cultures, which tended to form a loose, flat mat close to the surface of the oxygenation membrane (oriented on bottom of the HARV)^8^. Cell aggregation, as well as inclusion of microcarrier beads as a substrate for biofilm attachment, have both been used as proxies for biofilm formation in other bacterial LSMMG studies^9–13^. However, microscopic imaging of these biofilms is challenging, since the physical manipulation required to remove these biofilms from HARV vessels could introduce artifacts in biofilm structures. As an alternative, we describe herein a new method of growing bacterial biofilms under simulated microgravity in glass-bottomed 96-well plates on the random positioning machine (RPM). Because the bacteria constitutively express fluorescent proteins, biofilms can be directly imaged by confocal microscopy without further manipulation.

*S. mutans* and *Streptococcus gordonii* (a non-cariogenic resident of dental plaque biofilm) constitutively expressing green and red fluorescent proteins, respectively, were grown for 24 h as both single and dual species biofilms in wells of a glass-bottomed 96-well plate as described in Fig. 1. These biofilms were grown under conditions of 0 × g RPM (simulated microgravity), 0.9 × g RPM (the RPM setting closest to 1 × g, used as a control to account for RPM movement and vibrations), and 1 × g control (whereby the 96-well plate was grown statically in the same incubator housing the RPM). These biofilms were directly imaged by using a confocal microscope with an inverted stage, without further washing or other manipulation. Three-dimensional (z-stack) images (400x magnification) of these biofilms (Fig. 2) revealed that *S. mutans* 0 × g RPM biofilms underwent significant qualitative structural changes (decreased biovolume, increased formation of tower-like structures, less compact/less dense biofilms) relative to the normal gravity (1 × g) control and 0.9 × g RPM biofilms. In contrast, *S. gordonii* single-species 0 × g RPM biofilms displayed increased biovolume with extensive chaining of cells, relative to its corresponding 1 × g control and 0.9 × g RPM biofilms. The biofilm architecture of *S. mutans* 0 × g RPM biofilms could also be observed macroscopically (Supplementary Fig. 1). Both *S. mutans* and *S. gordonii* single species biofilms grown at simulated 0.9 × g closely resembled their respective static 1 × g biofilm controls. Quantification of biofilm biovolumes (Fig. 3) corroborated these qualitative observations: *S. gordonii* 0 × g single species biofilms contained ~10-fold greater biovolume compared to the *S. mutans* 0 × g single species biofilms, whereas 0.9 × g and 1 × g *S. mutans* single species biofilm biovolumes were ~2.5-3 fold larger than the corresponding *S. gordonii* biofilms (Fig. 3A). Within the 0 × g and 1 × g dual-species biofilms, the *S. mutans* biovolume appeared to outnumber *S. gordonii* biovolume by ~3:1, whereas approximately equal biovolumes of each species were measured in the 0.9 × g dual-species biofilms (Fig. 3B).

**Fig. 1:**
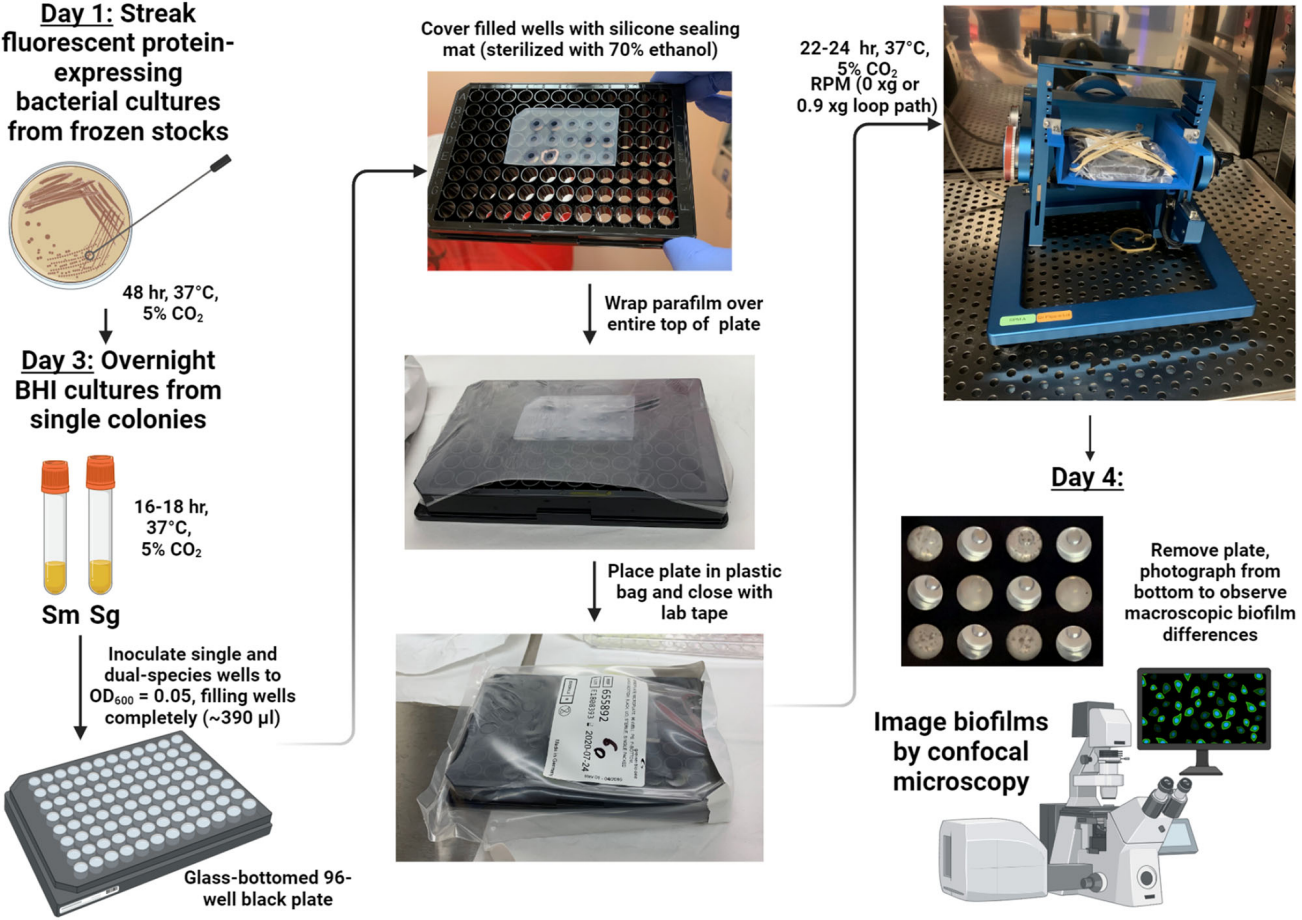
Experimental overview of RPM biofilm method. *S. mutans* and *S. gordonii* are each diluted from overnight cultures to an OD_600_ = 0.05 in biofilm medium containing sucrose and glucose and grown as single or dual species biofilms in wells of a glass-bottomed 96-well plate containing zero headspace and sealed with a silicone mat. After 22-24 h growth at 37 °C and 5% CO_2_, biofilms are directly imaged by using a confocal microscope with an inverted stage, without further washing or other manipulation. Image created with BioRender.com.

**Fig. 2:**
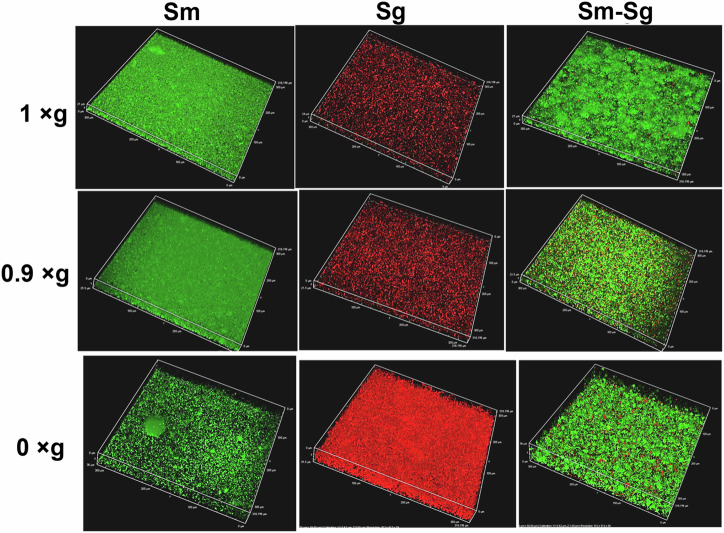
Biofilm experiments comparing RPM 0 × g, RPM 0.9 ×g, and static (1 × g) control growth conditions. *S. mutans* (Sm) and *S. gordonii* (Sg) were grown as single or dual-species biofilms for 22–24 h in sealed 96-well glass-bottomed plates as described in Fig. 1. Biofilms were imaged directly using an inverted confocal microscope at 400x magnification (green = Sm and red = Sg). Data representative of at least *n* = 3 experiments per strain per growth condition.

**Fig. 3:**
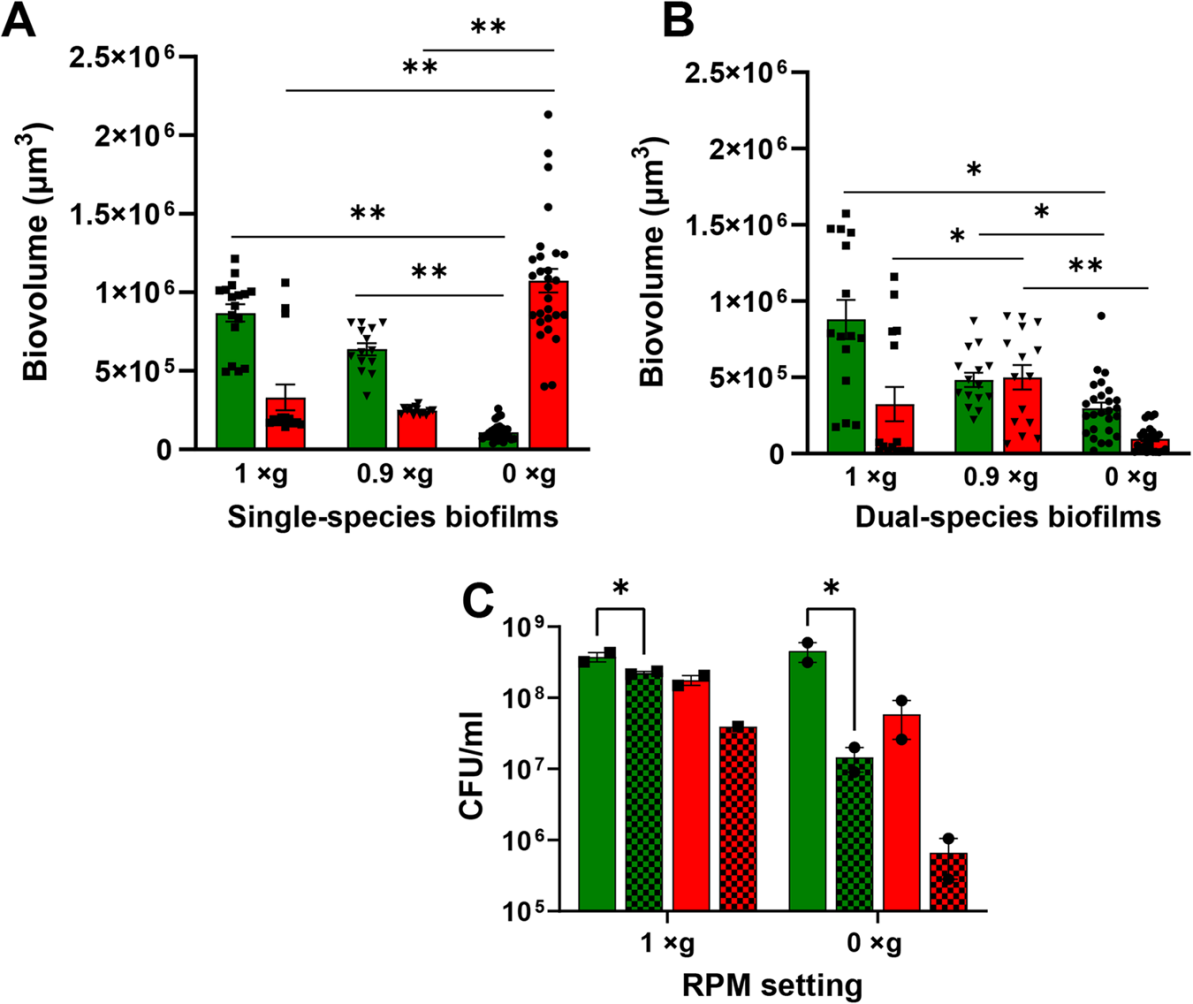
Biofilm biovolume and viability measurements. Confocal z-stack images of *S. mutans* (green) and *S. gordonii* (red) single species (**A**) and dual-species (**B**) biofilms were analyzed for biovolume using Nikon NIS-Elements Imaging Software. Data represents the average of 13–28 random fields of view acquired over at least *n* = 3 experiments. Error bars = standard error of the mean (SEM). One-way ANOVA followed by Dunn’s post-hoc test (*P < 0.05, **P < 0.01) was performed on each of the following biovolume comparisons: Single-species *S. mutans* (0 × g, 0.9 × g, 1 × g), single-species *S. gordonii* (0 × g, 0.9 × g, 1 × g), dual-species *S. mutans* (0 × g, 0.9 × g, 1 × g), dual-species *S. gordonii* (0 × g, 0.9 × g, 1 × g). **C:** Viability (CFU/ml) of *S. mutans* (green) and *S. gordonii* (red) single species attached biofilms (checker filled bars) and total biofilm wells (attached + planktonic; solid filled bars) was also measured. Data represents *n* = 2 independent experiments. Error bars = SEM. *P < 0.05 (paired t-test).

Single species *S. mutans* and *S. gordonii* biofilms were also grown at 1 × g with swapped reporter plasmids (Supplementary Fig. 2). These results demonstrated that *S. mutans* and *S. gordonii* expressing dsRed and sGFP, respectively, displayed similar patterns of biofilm formation as were observed in Fig. 2. However, dsRed fluorescence appeared to be brighter in *S. gordonii* biofilms relative to *S. mutans*, whereas sGFP fluorescence was comparable in both species. These results suggest the need for further optimization of the fluorescent reporters used in dual-species biofilm experiments, to ensure that fluorescent reporters are comparably bright and are not imparting effects on fitness. The viability of attached biofilms as well as total cell growth in each well was also assessed by CFU counts in 1 × g and 0 × g RPM cultures (Fig. 3C). These results showed that overall cell viability (comparing total well growth CFUs) was comparable for both species between the 1 × g and 0 × g RPM growth conditions. Additionally, both *S. mutans* and *S. gordonii* had less attached biofilm CFUs during 0 × g RPM growth relative to 1 × g growth. However, these CFU counts may not accurately reflect the biovolumes observed by confocal microscopy, as cell clumping and/or growth of cells in chains (in the case of *S. gordonii* 0 × g biofilms) may underestimate the actual number of cells.

The decreased biovolume of *S. mutans* single species 0 × g RPM biofilms correlates with previous *S. mutans* simulated microgravity studies using diamagnetic levitation^14^, in which *S. mutans* displayed a thinner but denser biofilm architecture^15^. *Staphylococcus aureus* also exhibited decreased auto aggregation (proxy for biofilm formation) when grown under simulated microgravity using an RPM^16^. In contrast, *S. gordonii* single species 0 × g RPM biofilms yielded increased biovolume (as assessed by CLSM) compared to their *S. mutans* biofilms counterparts, suggesting that not all oral streptococci respond to simulated microgravity in the same manner. In fact, several other bacterial species display increased biofilm biomass when grown under simulated microgravity. For example, *Bacillus subtilis* cultures pre-grown in LSMMG displayed increased biofilm when regrown at 1 × g^17^. *Escherichia coli*^9,10^, *S. aureus*^11^, *Klebsiella pneumoniae*^12^, and *Pseudomonas aeruginosa* biofilms^13^ were also increased when grown under LSMMG. *P. aeruginosa* biofilm formation was also assessed in spaceflight, conducted in a fluid processing apparatus (FPA) aboard two shuttle flights. These biofilms displayed increased biofilm biomass and thickness and a unique “column-and-canopy” structure relative to normal gravity controls^18^. These studies illustrate the importance of studying biofilm properties in response to simulated microgravity and partial gravities of interest, as different bacterial species of importance to astronaut health appear to respond differently to this environmental variable, potentially affecting their persistence and pathogenic potential during space flight missions.

Astronauts are currently subjected to rigorous preflight dental screening and treatment^19^. However, in the face of future long-term space flight missions, it is critical to understand how oral microbiota respond to microgravity and other space flight conditions, so that appropriate methods for maintaining dental health can be developed^19^. Both *S. mutans* and *S. gordonii* are facultative anaerobes found in the human oral cavity, with *S. mutans* a cariogenic early colonizer of dental plaque and *S. gordonii* a commensal/non-cariogenic member of this consortium. These species compete for nutrients and real estate within dental plaque, using mechanisms such as acidification and bacteriocin production (*S. mutans*) and H_2_0_2_ production (*S. gordonii*)^20–22^. Our RPM biofilm model results demonstrate that *S. gordonii* biofilm increased in simulated microgravity (Figs. 2 and 3**)**. Whether this translates to other non-cariogenic streptococci (such as *S. sanguinis* and other caries-inhibiting streptococcal species^23,24^), and their abilities to inhibit *S. mutans* growth in mixed species biofilms, will be the subject of future investigations.

Overall, these experiments demonstrate that this RPM biofilm growth model, combined with confocal microscopy imaging, is a non-invasive technique to study the effects of simulated microgravity on bacterial biofilms, with minimal architecture disruption due to the absence of washing/harvesting steps. In the case of oral streptococcal interactions, further optimization and validation of fluorescent reporters as accurate proxies for estimating biovolume (especially in dual-species biofilms) will need to be performed. This model also has widespread potential for studying the effects of simulated microgravity on biofilm architecture in other bacteria, assuming that they can be transformed to express constitutive fluorescent proteins and that they do not require highly aerobic growth conditions.

## Methods

### Streptococcal strains and culture conditions

*S. mutans* UA159 (constitutively expressing superfolder GFP in pDL278) and *S. gordonii* DL1 (constitutively expressing DsRed-Express2 in pDL278) were used in all experiments, previously described in^25^ and obtained from Dr. Robert Shields (Arkansas State University). For each biofilm experiment, strains were streaked for single colony isolation from −80 °C stocks (40% (v/v) glycerol) onto Brain Heart Infusion (BHI) agar plates containing 1 mg/ml spectinomycin. After 48 h of growth at 37 °C and 5% CO_2_, a single colony of each strain was inoculated from these plates into culture tubes containing BHI broth + 1 mg/ml spectinomycin and grown statically for 16-18 h at 37 °C and 5% CO_2_.

### Inoculation and setup of RPM biofilms

The overall methodology and experimental design are presented in Fig. 1. All steps of biofilm inoculation and setup were performed in a class 2AII biosafety cabinet to maintain sterility. For single-species biofilms, each overnight culture was diluted to an OD_600_ = 0.05 in semi-defined biofilm-promoting medium^26^ containing 10 mM sucrose, 11 mM glucose, and 500 µg/ml spectinomycin. For dual-species biofilms, overnight cultures of *S. mutans* and *S. gordonii* were each diluted to an OD_600_ = 0.025 into the same tube of biofilm medium. Diluted inoculums were vortexed for 10 sec, and ~390 µl aliquots were immediately transferred to replicate wells of a 96-well sterile glass-bottomed sensoplate (Greiner Bio-One), as this volume consistently yielded zero head space in the wells. A silicone micro-mat (cut to a size that covered all wells filled with inoculum) (Thermo Scientific) was sterilized with 70% (v/v) ethanol, dried completely, and used to seal the inoculum-filled wells. After ensuring there were no leaks or major air bubbles in the wells, each plate was wrapped in parafilm and placed in a plastic sealed bag prior to placing it on the RPM. All RPM experiments were conducted using Airbus RPM 2.0 instruments housed at the Kennedy Space Center Microgravity Simulation Support Facility (KSC-MSSF). Plates were grown on RPMs using the “partial G motion mode” set for either a 0 × g or a 0.9 × g path for 22-24 h at 37 °C and 5% CO_2_. As a 1 × g (normal gravity) control, biofilm plates were grown statically in the same incubator for the same period.

### Confocal microscopy and analysis of biofilm images

After growth, biofilms were immediately imaged using an inverted stage Nikon A1R point scanning confocal microscope with a Plan Fluor 40x Oil DIC H N2 lens. Green fluorescence was detected with 488 nm excitation and 535 nm emission, and red fluorescence was detected with 561 nm excitation and 595 nm emission. At least *n* = 3 independent experiments were conducted for both single species and dual species biofilms at 1 × g (normal gravity), simulated 0.9 × g (RPM), and simulated 0 × g (RPM), with *n* = 13-28 total random fields of view acquired for each bacterial species and biofilm growth condition. Biofilms were analyzed for biovolume using Nikon NIS-Elements Imaging Software, followed by statistical analysis using Sigmaplot 14.

### Quantification of biofilm cell viability

For CFU determinations, parallel 0 ×g RPM and 1 ×g biofilm cultures were grown as described above (duplicate wells per strain). After approximately 20.5 h growth, the silicone sealing mat was carefully removed from each 96-well plate. The entire content of one set of wells was harvested by scraping and vigorous pipetting to collect both attached and planktonic cells (“total biofilm well”). The duplicate set of wells was used to enumerate viable cells in the attached biofilm, whereby the culture supernatant (containing planktonic bacteria) was removed, an equal volume of sterile media was added, followed by harvesting of attached cells by scraping and vigorous pipetting (“attached biofilm well”). Statistical analysis was performed using Graphpad Prism 10.

## Supplementary information

Supplemental Figures

## Data Availability

All data presented in this manuscript is available in the presented figures.
