# Supplementary material for: Brief Communication: Confocal microscopy of oral streptococcal biofilms grown in simulated microgravity using a random positioning machine
Source: NPJ Microgravity. 2024 Sep 9;10:89. doi: 10.1038/s41526-024-00427-y (PMC11385976; doi:10.1038/s41526-024-00427-y)
Supplement: Supplementary file 1 — Supplemental Figures [file 41526_2024_427_MOESM1_ESM.pdf]

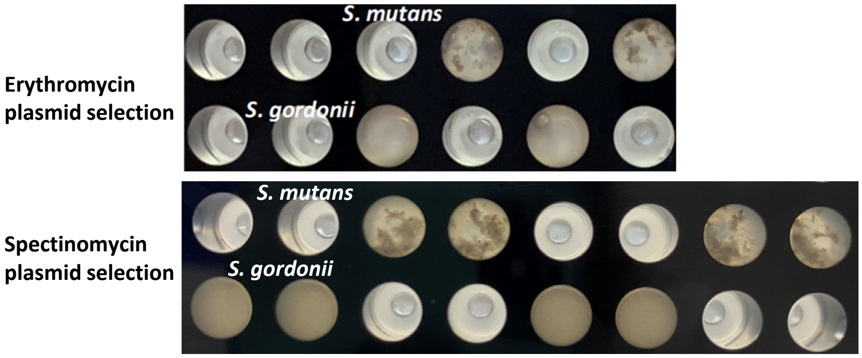

Supplemental Figure 1. *S. mutans* (GFP) and *S. gordonii* (dsRed) harboring fluorescent reporter plasmids requiring erythromycin selection (10 ug/ml; pOri23) or spectinomycin selection (500 ug/ml; pDL278) were grown as single species biofilms for approximately 20 hours in sealed 96-well glass-bottomed plates under 0 ×g RPM conditions as described in Figure 1. Macroscopic biofilms were photographed from the bottom of each 96-well plate. Data is representative of at least n=3 independent experiments. These results show comparable biofilm structures in the presence of two different antibiotics, suggesting that the antibiotic itself is unlikely having an impact on biofilm structure.

Sm 1 ×g **Sg ×g**

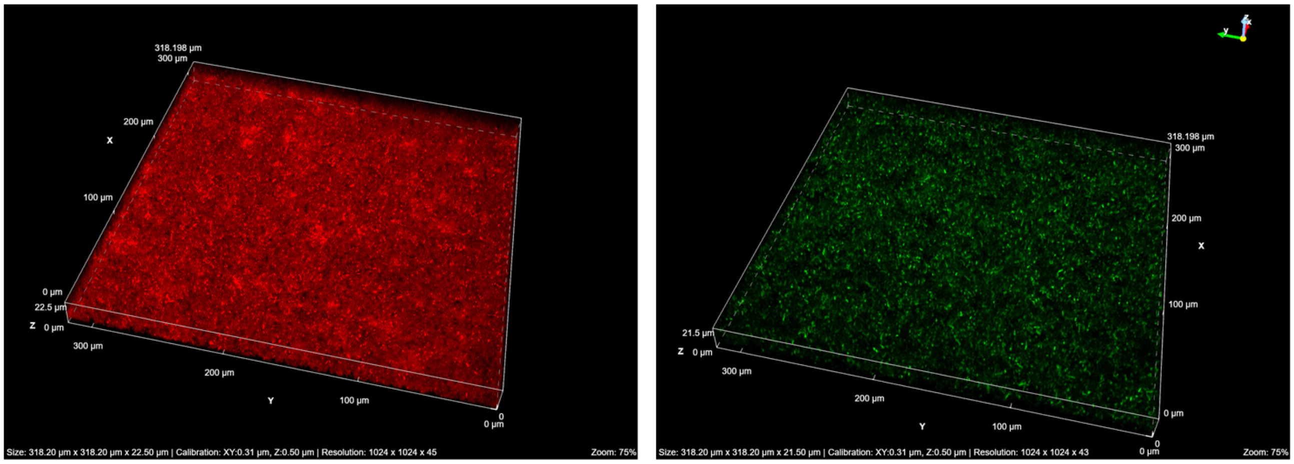

Supplemental Figure 2. Single species 1 ×g biofilms with swapped fluorescent reporters. Confocal z-stack images of *S. mutans* (red) and *S. gordonii* (green) single species biofilms grown for approximately 16 hours in sealed 96-well glass-bottomed plates under 1 ×g conditions as described in Figure 1. Biofilms were imaged directly using an inverted confocal microscope at 400x magnification (red = Sm and green = Sg). Data representative of n=2 independent experiments per strain.
